# Psychological Determinants of Conflict with the Law and Susceptibility to Rehabilitation in Relation to the Presence of Symptoms of Attention Deficit Hyperactivity Disorder

**DOI:** 10.3390/brainsci15020141

**Published:** 2025-01-31

**Authors:** Agnieszka Nowogrodzka, Mirosław Andrusiewicz, Ewa Mojs

**Affiliations:** 1 Department of Penitentiary Science, Academy of Justice, Wiśniowa 50, 02-520 Warszawa, Poland; 2 Department of Cell Biology, Poznan University of Medical Sciences, Rokietnicka 5D, 60-806 Poznań, Poland; andrus@ump.edu.pl; 3 Department of Clinical Psychology, Poznan University of Medical Sciences, Bukowska 70, 60-812 Poznań, Poland; ewamojs@ump.edu.pl

**Keywords:** attention deficit hyperactivity disorder (ADHD), criminal behavior, trauma, mental health, personality

## Abstract

**Background/Objectives:** Many prison-sentenced individuals exhibit symptoms of mental dysfunctions, including attention deficit hyperactivity disorder (ADHD). The presence of co-occurring mental disorders further complicates their rehabilitation and social reintegration efforts. Given these challenges, understanding the role of specific disorders, such as ADHD, is critical for developing targeted interventions tailored to the needs of incarcerated individuals and improving their outcomes. This research aimed to clarify the relationships among hyperactivity, criminal behavior, and psychological functioning to inform preventative and therapeutic strategies. **Methods:** This study investigated the complex interplay among attention deficit hyperactivity disorder (ADHD) symptoms, criminal behavior, and various psychological factors in a sample of 391 male inmates from low-security Polish prisons and a control group of non-offending men. Principal component analysis (PCA) and Spearman’s rank correlation were used to analyze the relationships among ADHD severity, type of crime (no crime, property crime, crime involving aggression), family functioning, childhood trauma, early maladaptive schemas, and mental health disorders. **Results:** The results revealed that while traumatic experiences were present across all groups, stronger family cohesion and support were associated with the absence of ADHD symptoms and criminal behavior. As ADHD severity and criminal behavior escalated, particularly in cases involving aggression, family support diminished, and maladaptive schemas, including “disconnection and rejection” and “excessive vigilance and inhibition”, became more prominent, alongside increased correlations with mental health issues (anxiety and depression). **Conclusions:** The findings underscore the crucial role of family environment and early intervention in mitigating the risks associated with ADHD and criminal behavior, highlighting the need for comprehensive interventions targeting maladaptive schemas and providing support for both internalizing and externalizing symptoms. Limitations include the retrospective nature of data collection and the exclusive focus on male inmates in low-security facilities.

## 1. Introduction

Global studies on the mental health of incarcerated individuals reveal that this group is disproportionately composed of socially marginalized individuals who frequently experience mental disorders [1]. Data from Canadian national studies indicate that up to 44% of incarcerated individuals exhibit symptoms of mental disorders [2]. Similarly, findings from Scandinavian penitentiary institutions suggest that this figure ranges between 50% and 75%, while U.S. prison statistics report that approximately 70% of juvenile offenders exhibit at least one mental disorder [3,4]. Furthermore, incarcerated individuals are more likely to be diagnosed with co-occurring mental disorders compared to the general population [5]. These clinical symptoms complicate their ability to engage in social rehabilitation and learn new behaviors and coping mechanisms [6,7]. This presents a significant challenge for professionals tasked with supporting and preparing them for reintegration into society. Consequently, research aimed at identifying mental disorders among inmates is vital for developing targeted interventions for this population.

Attention deficit hyperactivity disorder (ADHD) is a neurodevelopmental disorder typically diagnosed in childhood around the ages of 6–7. It is estimated that ADHD symptoms are observed in 5.9% to 10% of the general population [8,9,10,11]. Although symptoms often diminish with age, they can persist into adolescence and adulthood to varying degrees. As a result, there is increasing interest in understanding the transition from childhood ADHD to adult ADHD, a progression supported by long-term studies [12,13]. In adulthood, the prevalence of ADHD is estimated to range between 3% and 6% [14,15], though symptoms persist into adulthood in 50% to 70% of cases [16,17,18].

Research indicates that the deficits associated with ADHD can significantly impact the long-term functioning of affected individuals [19]. Compared to the general population, people with ADHD are more likely to experience mental and behavioral disorders [20]. Traits such as neuroticism and impulsivity are intrinsic to the disorder, often manifesting as emotional regulation deficits, distractibility, irresponsibility, and risk-taking tendencies in later stages of life [21]. Among adults with ADHD, conduct disorders and personality disorders are commonly diagnosed [22,23,24]. Additionally, up to 12% of adults with ADHD are diagnosed with anxiety disorders, and a significant proportion also experience depressive symptoms [25,26,27]. Addiction is another disorder frequently observed in adults with ADHD [28,29,30,31].

Attention deficit hyperactivity disorder has been studied for its impact on various aspects of individual behavior, particularly in the context of criminal behavior. Numerous studies have indicated a significant correlation between ADHD symptoms and an increased risk of engaging in criminal activities [32,33,34,35,36,37]. It was suggested that individuals with ADHD, particularly those who were not diagnosed early or who did not receive appropriate interventions, were more likely to exhibit impulsive behaviors that can lead to criminal actions, especially during adolescence [32,33,34].

Several studies, including both short- and long-term analyses conducted among offender populations [32,35,36,38], suggest that ADHD is a risk factor for criminal behavior. For example, research conducted on Swedish prisoners serving long-term sentences found that up to 40% of inmates could be diagnosed with ADHD [35]. However, it is important to note that the incarcerated individuals with ADHD in this study more frequently exhibited symptoms of other mental disorders compared to a control group of non-incarcerated individuals with ADHD. Similarly, a meta-analysis by S. Young et al. [36] based on data from 15 countries estimated the prevalence of ADHD among the prison population to be approximately 25.5%, highlighting that individuals with ADHD constitute a significant portion of the incarcerated population.

In Danish population-based studies, the relationship between childhood or adolescent ADHD and criminal convictions was examined, with additional factors such as co-occurring mental disorders and parental socioeconomic status considered as potential aggravating influences. These studies indicated that individuals with ADHD were 1.6 times more likely to have criminal convictions, a figure notably lower than most similar analyses [39].

Findings from a study conducted in a Norwegian prison in Bergen are also noteworthy [40]. This study analyzed 43 inmates referred for psychiatric treatment through the penitentiary healthcare system. Depending on the diagnostic tools used, ADHD symptoms were identified in 74% to 100% of these inmates. However, in-depth studies and additional interviews with third parties reduced this estimate to 35%. It is worth noting that this group was referred for consultation based on observable deficits identified by their environment, indicating that they represented a subgroup at high risk of mental disorders within the inmate population.

The psychological underpinnings of this relationship are complex. Studies by Moffitt et al. [41], replicated across various populations, including American and British samples [42], indicate that attention deficits are primarily associated with educational difficulties, while impulsivity and hyperactivity increase the likelihood of engaging in conflicts with the law. Moreover, as noted by the authors of both studies, the functioning of individuals with ADHD is significantly influenced by factors such as socioeconomic conditions (particularly during childhood), the school environment, and the presence of mature parenting during adolescence. According to the cited research, positive behavioral changes can be fostered within this group at any stage of development.

**Figure 1:**
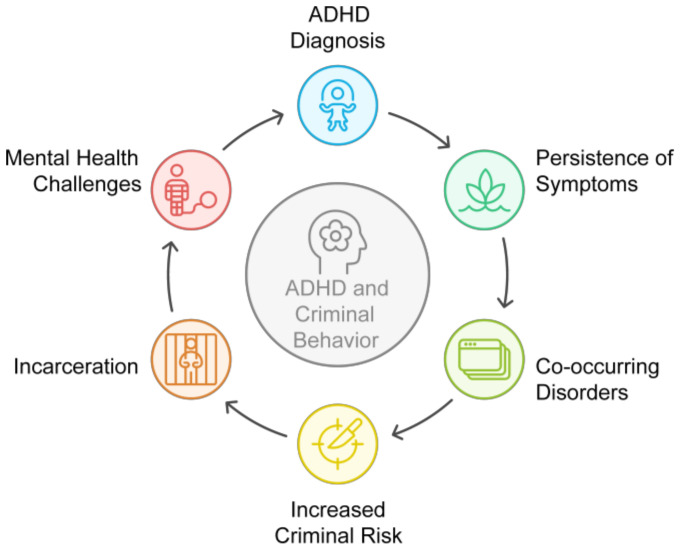
The cycle of ADHD and criminal behavior. This diagram illustrates the interconnected factors contributing to the increased risk of criminal behavior in individuals with ADHD, emphasizing how various elements reinforce one another, creating a challenging pathway.

Additionally, maladaptive schemas rooted in early negative experiences or trauma can further exacerbate the risk of criminal behavior in individuals with ADHD [43,44,45,46]. Studies have demonstrated that childhood trauma, including abuse and neglect, is prevalent among individuals with ADHD and contributes to the development of maladaptive schemas that shape subsequent behaviors [46,47,48,49]. These findings underscore the importance of understanding these psychological factors when analyzing the pathways linking ADHD to criminal behavior (Figure 1).

Long-term studies comparing three groups of children diagnosed with ADHD in hospital settings provide further insight [32,50]. These groups included children with ADHD alone, children with ADHD and oppositional defiant behaviors, and children with ADHD and conduct disorders. A control group was also included for comparison. Observed until at least age 18, all ADHD-diagnosed groups committed crimes more frequently than the control group, with the worst outcomes seen in children with both ADHD and conduct disorders, who committed crimes almost nine times more often than controls.

To summarize, most studies on ADHD and criminal behavior suggest that individuals with ADHD who also experience other mental disorders are more prone to criminal activity. Thus, ADHD alone is not considered a direct risk factor for criminal behavior. Instead, long-term studies often focus on clinical groups with pronounced symptoms or problematic behaviors, which may not represent the broader population of individuals with ADHD [51]. Criminal behavior is more prevalent among individuals with ADHD when additional deficits or disorders—such as improper socialization, socioeconomic challenges, or conduct disorders and addictions—are present. When ADHD coexists with these factors, the risk of criminal behavior significantly increases [28,51,52]. S. Barra et al. [53] also emphasize that ADHD is frequently accompanied by significant emotional and behavioral issues not captured by diagnostic criteria. These issues can influence the functioning and behavior of individuals with ADHD, including their involvement in criminal activity, even when not directly related to the disorder.

One category of crimes committed by this group includes traffic offenses. The coexistence of ADHD with personality traits such as impulsivity, hyperactivity, and an increased tendency to consume alcohol heightens the likelihood of uncontrolled and dangerous driving behaviors [54,55,56].

Despite the growing literature data, there remain significant gaps in understanding how ADHD symptoms interact with psychological factors such as maladaptive schemas and childhood trauma to influence specific types of crime. For instance, while ADHD is widely associated with impulsivity and aggressive behavior, the nuanced relationship between ADHD symptoms and the underlying psychological factors often receive insufficient attention [54,55,56]. This lack of clarity impedes the development of targeted intervention strategies that could mitigate the risk of criminal behavior in affected individuals. To address these gaps, this study proposes the following specific hypotheses: (H1) individuals with higher levels of maladaptive schemas are more likely to exhibit criminal behavior compared to those with adaptive schemas; (H2) a history of childhood trauma is positively correlated with the severity of ADHD symptoms and criminal behavior; and (H3) the type of crime committed varies depending on the individual’s psychological profile and ADHD symptomatology. By exploring these hypotheses, this research aims to enhance the understanding of the interplay among ADHD, criminal behavior, and psychological factors in penitentiary contexts, ultimately contributing to the development of more effective psychosocial support programs and intervention strategies.

This study aimed to analyze the relationship between hyperactivity and criminal behavior among first-time and repeat offenders serving sentences in low-security prisons characterized by a lower degree of moral degradation. A control group of non-offending men was included for comparison. In the comparisons, we considered varying levels of ADHD symptoms in the studied groups, as well as the different types of crimes committed by the offenders (property crimes and crimes involving aggression). This approach allowed for a better understanding of the connections among family relationships, early childhood trauma, maladaptive emotional schemas, and mental disorders.

## 2. Materials and Methods

### 2.1. Participants

The study included detainees from semi-open facilities in Poland selected for presenting a lower degree of moral degradation. Individuals from these facilities were included, excluding those with intellectual disabilities. Of 437 invited, 411 responded, and 350 questionnaires were analyzed. The choice of semi-open facilities was justified by the relevance of this subgroup in the Polish prison system, characterized by lower security and greater compliance of detainees with rules. Individuals incarcerated in these units are generally those deemed to have a lower degree of moral degradation, serving sentences for crimes of lesser social harm or demonstrating good behavior and compliance with prison regulations. These inmates typically exhibit greater independence and adaptability in their interactions with others.

Semi-open correctional facilities operate with reduced security measures, such as open cells during the day and permitting inmates to work outside the prison under minimal supervision or in some cases without any escort [57]. Inmates in these settings are often more motivated to exhibit socially acceptable behavior. To underscore the significance of this group within the Polish penitentiary system, it is notable that according to 2023 statistics from the Polish Prison Service [58], there were 34,684 inmates in closed facilities at the end of the year compared to 28,129 in semi-open facilities. Inmates in semi-open facilities accounted for 43.8% of the prison population, while those in closed facilities comprised 54.09%, with the remaining 2.2% in open facilities. These statistics highlight the diversity and complexity of the incarcerated population, emphasizing the importance of studying distinct subgroups.

Despite this diversity, research—particularly on ADHD—has predominantly focused on inmates in closed facilities with higher degrees of moral degradation. This tendency mirrors the generalization of findings from clinical groups with pronounced ADHD symptoms to the broader ADHD population, a practice that can lead to overinterpretation and limited applicability of results.

The study was conducted in accordance with the Declaration of Helsinki (2013) and approved by the Institutional Review Board of Poznan University of Medical Sciences, Poznan, Poland (398/2022, 19 May 2022).

### 2.2. Instruments and Procedures

The study was conducted in 2023 across two regions of Poland—eastern and western—in four different penitentiary units in Poznań, Iława, Wronki, and Białystok. This geographical spread was designed to increase the diversity of the study population. Participants were informed about the study’s purpose and assured of their responses’ anonymity. Written consent was obtained from all participants prior to their involvement, and participation was entirely voluntary.

The researchers administering the study received specialized training on using the DIVA-5.0 diagnostic interview and providing clear instructions for other self-report tools. This ensured consistency in the data collection process and accuracy in the application of the diagnostic methods.

The study utilized standardized or adapted questionnaire methods tailored to the Polish population. The researchers conducted the ADHD diagnostic interviews, while the remaining self-report measures were independently completed by participants using the “paper-and-pencil” method.

The primary tool for ADHD diagnosis was the Diagnostic Interview for ADHD in Adults (DIVA-5.0). This interview enables the identification of attention deficit hyperactivity disorder (ADHD) in adults. During the interview, the researchers asked participants questions about the diagnostic criteria and provided examples of ADHD-related symptoms observed in childhood and adulthood. The diagnostic criteria employed in this tool align with those outlined in the DSM-V. The descriptions of ADHD-related behaviors comprehensively address all diagnostic criteria. The current version of DIVA-5.0 is an evolution of its predecessor, DIVA 2.0 [59]. Generally, the tool’s sensitivity is reported at 91.30%, while its specificity is 93.62% [60]. Like others, the Polish version of the interview was developed by the DIVA Foundation. As with other language versions, the interview was first translated into Polish and then back-translated into Dutch.

Early childhood traumatic experiences were assessed using the Childhood Trauma Questionnaire (CTQ). This tool consists of 28 questions, which participants answer on a 1-to-5 Likert scale. The questionnaire enables the evaluation of early childhood trauma related to emotional, physical, and sexual abuse, as well as emotional and physical neglect [61,62]. The study used the Polish version of the tool adapted by Murzyn [63]. The original tool demonstrates satisfactory psychometric parameters regarding reliability (α = 0.79–0.96) [62].

The Young Schema Questionnaire (YSQ-S3; Polish translation) was used to measure early maladaptive emotional schemas [64]. Young identified eighteen persistent behavioral, emotional, and motivational patterns presented by individuals. The questionnaire allows for evaluating the intensity of each of these patterns, as well as the specific areas they cover. Participants responded to 90 statements using a Likert scale (range: 1 to 6). The tool was adapted in 2018. Reliability analysis using Cronbach’s alpha revealed varied internal consistency across subscales, ranging from 0.62 to 0.81. These values can be considered acceptable and comparable to similar validations of this tool in other countries. The Family Assessment Scale (FAS; adapted and standardized for Polish conditions in 2013) [65] was used to measure participants’ functioning patterns in the family environment. The questionnaire consists of 62 questions, to which participants respond by selecting one answer on a 1-to-5 Likert scale. The statements form eight scales that provide a comprehensive description of the family. The reliability coefficients for the Polish version range from 0.70 to 0.93 (Cronbach’s alpha). Another tool used in the study was the General Health Questionnaire (GHQ-30; Polish translation) for assessing the mental health of adults in the general population [66]. The tool was adapted to Polish conditions in 2010. It can be used to detect non-psychotic mental disorders. The total test score includes factors such as depression and anxiety, interpersonal relationships, and overall functioning disorders. Participants respond to 30 questions using a four-point Likert scale. The scale’s internal consistency was evaluated using Cronbach’s alpha, which reached 0.97, indicating high homogeneity. The survey was also used to gather basic data about the participants for the study.

### 2.3. Preliminary Analyses

This study explored the relationships between family functioning, childhood trauma, early maladaptive schemas, mental health disorders, ADHD severity, and criminal behavior in a sample of 391 males. The sociodemographic data of all participants are presented in Table 1. The assessment included eight measures of family functioning (“general assessment”, “sustainable coherence”, “sustainable flexibility”, “unbound”, “tangled”, “stiffness”, “chaotic”, “family communication”, and “satisfaction with family life”), five measures of childhood traumatic events (“physical neglect”, “emotional neglect”, “sexual abuse”, “physical abuse”, and “emotional abuse”), five measures of early maladaptive schemas (“disconnection and rejection”, “weakened autonomy”, “defective borders”, “targeting others”, and “excessive vigilance and inhibition”), and three mental health disorders (“anxiety and depression”, “disturbed interpersonal relationships”, and “impairment of general functioning”). After excluding 41 outliers, the analyses focused on two primary divisions: ADHD severity (non-ADHD, moderate ADHD, severe ADHD; *n* = 301, 32, and 17 respectively) and criminal behavior (no criminal record, property crime, crime involving aggression; *n* = 118, 110, and 122 respectively). Principal component analysis (PCA), using Statistica 13, was conducted separately on two sets of variables: (1) thirteen variables encompassing family functioning and childhood trauma and (2) eight variables related to maladaptive schemas and mental health disorders. Correlation matrices based on correlation analysis with variances calculated as SS/(N-1) were used as input for PCA. A scree plot determined the number of principal components to retain. Spearman’s rank correlation test assessed the strength of relationships among the analyzed variables. The values of the correlation strength were interpreted as: <0.2—very weak; 0.20 to 0.39—weak; 0.40 to 0.59 –moderate; 0.60 to 0.79—strong; and 0.80 to 1.00—very strong. A *p*-value of <0.05 was considered significant.

In statistical analysis, our choice of principal component analysis (PCA) was appropriate for reducing the dimensionality of our data and identifying the underlying structure of the variables associated with ADHD and criminal behavior. It was particularly beneficial in dealing with high-dimensional data, as it simplifies analysis and visualization without losing critical information. PCA enabled the visualization of high-dimensional data in two dimensions. We extracted the principal components that accounted for the greatest variance, enhancing the interpretability and effectiveness of those analyses.

## 3. Results

We used principal component analysis (PCA) to examine the relationships among ADHD severity (non-ADHD, moderate, severe), criminal behavior (no record, property crime, crime involving aggression), and various psychological and behavioral variables. Based on the pattern matrix of factor loadings, the PCA identified principal components explaining significant variance within the dataset. Each component represents a linear combination of the original variables, revealing the underlying structure of the data. The results provide insights into the factors associated with ADHD severity and its relationship to criminal behavior. Biplots visualize the relationships between variables and components. The proximity of variables within each plot suggests correlations; for example, the consistent proximity of “anxiety” and “depression” would indicate a strong correlation. Furthermore, the clustering of data points based on ADHD severity and crime type reveals potential distinct patterns associated with different levels of the disorder and types of criminal activity. Points that are close together within a quadrant indicate a strong correlation or similar loading on the principal components. These variables tend to behave similarly or share common underlying factors. Different clusters or spread-out points suggest variability among the variables, indicating distinct influences or contributions to the principal components. The axes represent the principal components (PC1, PC2), which are linear combinations of original variables. The percentage explains how much variance each component accounts for in the data. Quadrant I (top right): variables in this quadrant positively contribute to the axes’ principal components. Quadrant II (top left): variables here contribute positively to one component and negatively to the other. Quadrant III (bottom left): variables in this quadrant negatively affect both components. Quadrant IV (bottom right): variables positively contribute to one component, but negatively to the other.

### 3.1. Family Functioning and Early Childhood Traumatic Events

**Figure 2:**
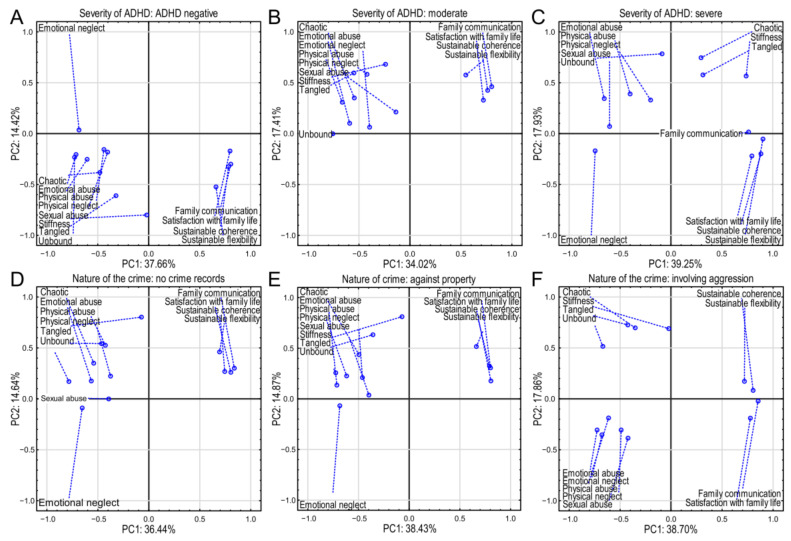
Principal component analysis (PCA) of family environment factors and their relationship to ADHD severity and criminal behavior. Six biplots display the relationships between family environment variables and principal components (PC1 and PC2) for six groups: three levels of ADHD severity (negative, moderate, severe; panels (**A**–**C**)) and three types of criminal behavior (no crime, crime against property, crime involving aggression; panels (**D**–**F**)). Each point represents a variable. The closer a point is to an axis, the higher the correlation (positive or negative) with that PC. The percentage of variance explained by each PC is shown. The dashed lines connect the variables to their coordinates.

Three separate principal component analysis plots were constructed relating to the severity of ADHD in different categories: ADHD-negative, moderate, and severe (Figure 2A–C). In ADHD-negative cases, principal component 1 explains 37.66% of the variance and 2 14.42% (Figure 2A). Emotional abuse (−0.713), emotional neglect (−0.685), and unbound (−0.727) indicate notable negative environmental impacts (Supplementary Materials Table S1). The variables highlight adverse and severe experiences. Family communication (0.806) and satisfaction with family life (0.798) reveal a strong positive correlation, indicating significant family support. Both are strongly associated with Factor 1, indicating strong ties to positive family dynamics. Additionally, sustainable coherence (0.785) and flexibility (0.660) highlight adaptive family dynamics (Supplementary Materials Table S1).

The moderate-ADHD group principal component 1 explained 34.02% of the variance, and PC2 was 17.41% (Figure 2B). Strong negative impacts were emotional abuse (−0.714) and emotional neglect (−0.685). This indicates a similar pattern, suggesting that abuse is more strongly related to the moderate-ADHD group in comparison to positive family aspects (Supplementary Materials Table S1). “Unbound” (−0.724) and “emotional neglect” (−0.685) were of significant concern, reflecting potential internal struggles and past trauma. The variables showed how significant concern, reflecting potential internal struggles and past trauma, can influence ADHD patients. Positive influence revealed that family communication (0.803) and satisfaction with family life (0.807) could be interpreted as continued family support. Sustainable coherence (0.791) indicated some resilience (Supplementary Materials Table S1) and was more pronounced, showing the impact on this group of moderate negative experiences, with family dynamics still playing a supportive role.

In the severe-ADHD group, component 1 explained 39.25% of the variance and PC2 17.93% (Figure 2C). Negative factors such as emotional neglect (−0.744), physical abuse (−0.725), and emotional abuse (−0.656) indicated significant impacts of negative experiences. Positive influences of sustainable coherence (0.885) and flexibility (0.905) suggest high adaptability and possibly compensatory mechanisms for negative factors (Supplementary Materials Table S1). A notable change in “unbound” showed less negative correlation compared to previous tables, suggesting more complexity in behavior patterns within severe ADHD. Severe ADHD was associated more strongly with various abuse factors, but positive family dynamics also played a role. There was a strong influence of negative factors, indicating fewer protective effects from family.

Each plot in Figure 2A–C highlights a consistent theme where certain negative experiences are associated with different severities of ADHD, whereas family dynamics and satisfaction appear to be potential protective factors. Understanding these relationships can inform targeted interventions focusing on family communication and addressing abuse to potentially mitigate ADHD severity.

In the individuals without criminal records, principal component 1 explained 36.44% of the variance and principal component 2 14.87% (Figure 2D). Negative associations such as emotional neglect (−0.653) and unbound (−0.785) highlight potential areas of concern (Supplementary Materials Table S2). Still, emotional neglect was revealed to be less significant than in criminal groups. Physical neglect (0.559) and emotional neglect (0.517) values indicated that past neglectful experiences were associated even in individuals with no criminal records. Family communication (0.839) and satisfaction with family life (0.804) values showed that strong family support was associated with the absence of criminal behavior (Supplementary Materials Table S2). Individuals without criminal records benefit from strong family support, which offsets negative experiences like abuse or neglect. This points to the importance of supportive environments in preventing potential criminal behaviors. In the crimes-against-property group, principal component 1 explained 38.43% of the variance and PC2 14.87% (Figure 2E). Negative influence in crimes without aggression showed associations with emotional neglect (−0.685) and physical abuse (−0.617). This indicates a strong association with property crimes. Physical abuse (−0.617) and emotional abuse (−0.714) had significant negative loadings, emphasizing the severe impacts of trauma. Family communication (0.803) and sustainable coherence (0.791) maintained some protective effects (Supplementary Materials Table S2), suggesting that these factors may deter property crimes. Positive family dynamics can help reduce these criminal tendencies. Important negative factors, though less impactful in this group, indicate how neglect and abuse are linked to property crimes, with positive family dynamics providing some mitigation. In cases with criminal records involving aggression, principal component 1 explained 38.70% of the variance and PC2 17.86% (Figure 2F). Emotional abuse (−0.726) and emotional neglect (−0.679) were strongly correlated with aggressive behaviors. The influence of “unbound” and “tangled” indicate the complexities of aggression-related behaviors linked to ADHD problems, especially when compounded by negative experiences. Positive associations such as family communication (0.854) were significant mitigating factors for aggressive behavior and offered protective effects, though weaker in this context. Sustainable coherence (0.804) remained important, highlighting its role in moderating aggression (Supplementary Materials Table S2). Aggressive criminal behavior was closely related to various forms of abuse and temper issues, with family communication and emotional expression serving as mitigating factors. There was a strong correlation between negative experiences and aggressive behavior, with some protective family effects.

Across all groups, adverse experiences (e.g., neglect, abuse) correlated with criminal behaviors, while positive family interactions offered protective effects. Fostering supportive family environments and addressing abuse can reduce criminal tendencies, highlighting areas for potential intervention and prevention. Family support in communication and satisfaction served as protective factors across groups and were generally protective, but varied in influence across contexts, with consistently strong negative impacts of emotional and physical abuse/neglect, especially in more severe ADHD and criminal categories. Interventions should target these areas to mitigate effects, emphasizing the importance of addressing adverse experiences and enhancing family support to reduce unfavorable outcomes related to ADHD and criminal behavior. Thus, therapeutic interventions should reinforce the importance of strengthening family functioning and addressing traumatic experiences to reduce ADHD impact and criminal tendencies.

Spearman’s coefficient correlations also supported the principal component observations. The correlation matrices revealed exciting patterns in the relationships between family environment variables and the six groups (three levels of ADHD severity and three crime types) (Supplementary Materials: Tables S3 and S4). We observed negative correlations with positive family functioning: across all six groups, sustainable coherence, sustainable flexibility, and family communication showed strong positive correlations with one another and consistently exhibited strong negative correlations with unbound, chaotic, emotional neglect, emotional abuse, and physical abuse. This indicates that better family functioning (higher scores on positive variables) is associated with lower levels of family dysfunction (lower scores on negative variables). Unbound, chaotic, emotional neglect, emotional abuse, and physical abuse tended to correlate positively with one another, representing different facets of dysfunctional family environments. The magnitude of these correlations varied across the six groups, suggesting the relative prominence of these aspects differs according to ADHD severity and crime type. “Sexual abuse” showed a more variable pattern, sometimes correlating more strongly with other forms of abuse and sometimes more weakly.

ADHD severity and family environment correlations suggested a trend where higher ADHD severity (moving from negative to severe) is associated with a weakening of positive family factors (lower correlations with sustainable coherence, sustainable flexibility, and family communication) and a simultaneous strengthening of negative family factors (higher correlations with unbound, chaotic, emotional neglect, etc.). The patterns of the three crime-related groups suggest that the no-crime group shows stronger correlations with the positive family factors and weaker correlations with the negative factors than the groups with crimes, particularly those involving aggression.

The shift in correlations across ADHD severity groups suggests a possible dose–response effect, where more severe ADHD is associated with a more dysfunctional family environment. For instance, the negative correlations between positive family factors and negative factors might become less pronounced as ADHD severity increases. Regarding criminality, the no-crime group showed the strongest positive correlations with positive family factors and the weakest correlations with negative family factors. The crime-against-property group showed somewhat of a pattern between the no-crime and aggression groups, implying a possible gradient in the relationship between family environment dysfunction and type of criminal behavior. The correlation patterns showed a gradient effect related to the kind of crime committed. The no-crime group displayed the most substantial negative correlations between the positive family functioning variables and the negative variables.

In contrast, the “crime involving aggression” group showed much weaker negative correlations or sometimes even positive correlations between some positive and negative variables. This suggests that progressively more severe criminal behaviors are associated with less influence from positive family environment factors and perhaps even a more complex interplay between positive and negative family factors. The crime-against-property group showed patterns somewhat intermediate between the no-crime and aggression groups.

These correlation matrices support the hypothesis that family environment plays a significant role in both ADHD and criminal behavior. A positive family environment appears to have a protective effect against both conditions. However, the strength of this protective effect seems to diminish with increasing ADHD severity and the severity of criminal behavior. The results suggest a complex interaction where multiple factors related to both family environment and individual characteristics contribute to both ADHD presentation and engagement in criminal activity.

### 3.2. Early Maladaptive Emotional Patterns and Mental Health Disorders

**Figure 3:**
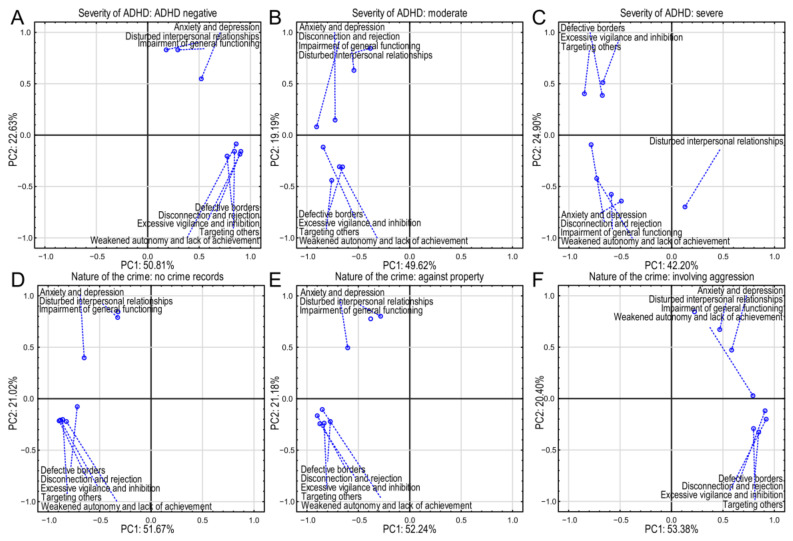
Principal component analysis (PCA) biplots illustrating the relationships among psychological and behavioral characteristics and ADHD severity and criminal behavior. Each panel displays the loading of variables on the first two principal components (PC1 and PC2). The percentage of variance explained by each PC is indicated. Points closer to the axes indicate stronger positive (positive side of the axis) or negative (negative side of the axis) correlations. Dashed lines connect each variable to its coordinate. (**A**–**C**) Associations of psychological and behavioral characteristics with different levels of ADHD severity. (**D**–**F**) Associations of psychological and behavioral characteristics with different types of criminal behavior.

Across all panels in Figure 3A–F, PC1 consistently accounts for a larger proportion of the variance (between 42.20% and 53.38%) than PC2 (between 19.19% and 24.90%). The exact percentages vary slightly across the panels, indicating that the relative importance of the first two principal components might be subtly influenced by the specific group (ADHD severity or crime type).

The non-ADHD group biplot shows that PC1 explains 50.81% of the variance, and PC2 explains 22.63% (Figure 3A). “Anxiety and depression”, “disturbed interpersonal relationships”, and “impairment of general functioning” (internalizing characteristics) are positioned on the negative side of PC2. “Weakened autonomy and lack of achievement”, “defective borders”, “targeting others”, “disconnection and rejection”, and “excessive vigilance and inhibition” (externalizing characteristics) are located more on the negative side of PC1. Strong positive loadings were observed for disconnection and rejection (0.906), weakened autonomy (0.861), defective borders (0.772), targeting others (0.844), and excessive vigilance (0.897) (Supplementary Materials Table S5). This suggests that PC1 represents externalizing behaviors and indicates these are key constructs for individuals without ADHD. Principal component 2 showed strong positive loadings for anxiety and depression (0.548), disturbed interpersonal relationships (0.830), and impairment of general functioning (0.829) (Supplementary Materials Table S5). This indicates that PC2 represents internalizing difficulties and suggests relationship challenges in the non-ADHD group. Disconnection and rejection showed a strong positive association, implying that individuals without ADHD are likely to experience significant feelings of disconnection and rejection. Excessive vigilance and inhibition had another strong positive loading, highlighting anxiety-driven behaviors. Although positive, the loading for disturbed interpersonal relationships was less significant compared to others, indicating less direct correlation within this factor. This grouping suggests that individuals without ADHD struggle primarily with feelings of rejection and anxiety, impacting their interpersonal relationships.

In the moderate-ADHD group, PC1 explained 49.62% of the variance and PC2 19.19% (Figure 3B). There were strong negative loadings in PC1 for disconnection and rejection (−0.905), weakened autonomy (−0.684), defective borders (−0.654), targeting others (−0.758), excessive vigilance (−0.843), and anxiety and depression (−0.727) compared to non-ADHD cases, indicating a slightly different impact with moderate severity (Supplementary Materials Table S5). This suggests that PC1 represents a combination of externalizing and internalizing problems with a negative association. On the other hand, in PC2, strong positive loading for disturbed interpersonal relationships (0.844) and impairment of general functioning (0.630) emphasizes relationship and functional impairments (Supplementary Materials Table S5). The strong negative loading for disconnection and rejection indicates that as individuals experience moderate ADHD symptoms, feelings of disconnection may manifest differently or become internalized, and that for anxiety and depression shows that anxiety becomes a significant aspect of their experience. Impairment of general functioning strongly correlated with factor 2, indicating that interpersonal relationships start to show significant impairment with moderate ADHD severity. This suggests that as ADHD symptom severity increases, individuals experience less overt disconnection, but greater internal struggles with anxiety and functioning.

In the severe-ADHD group (Figure 3C), PC1 explained 42.20% of the variance and PC2 24.90%. Principal component 1 revealed strong negative loadings for disconnection and rejection (−0.785), weakened autonomy (−0.729), defective borders (−0.675), targeting others (−0.850), and excessive vigilance (−0.669). Disconnection and rejection and excessive vigilance and inhibition again have high negative loadings, underscoring their continued importance (Supplementary Materials Table S5). Similarly to the moderate ADHD group, this points to externalizing issues, but with a negative association. PC2 revealed strong negative loading for anxiety and depression (−0.643) and disturbed interpersonal relationships (−0.699), critical issues in severe ADHD, suggesting a negative association with internalizing problems in this group (Supplementary Materials Table S5). Disconnection and rejection indicates a sustained negative experience, but hints at resilience in coping mechanisms. Anxiety and depression is moderately negative, showing these experiences are critical in severe cases, perhaps pointing to more profound emotional distress. Disturbed interpersonal relationships highlights significant relationship issues, suggesting that severe ADHD strongly impacts social connections, leading to potential conflicts or isolation. Here, the analysis indicates that in severe ADHD, emotional health and interpersonal dynamics are substantially impaired.

PC1 explained 51.67% of the variance in cases with no criminal records and PC2 21.02% (Figure 3D). PC1 showed strong negative loadings for disconnection and rejection (−0.894), weakened autonomy (−0.822), defective borders (−0.716), targeting others (−0.858), and excessive vigilance (−0.882) (Supplementary Materials Table S6). Similarly to ADHD, disconnection and rejection (−0.894) and excessive vigilance and inhibition (−0.882) were central to the factors affecting individuals without criminal records (Supplementary Materials Table S6). This shows a negative association with externalizing behaviors. Additionally, there were strong positive loadings for disturbed interpersonal relationships (0.790) and impairment of general functioning (0.844), indicating internalizing issues (Supplementary Materials Table S6). Impairment of general functioning had a strong loading in PC2, indicating significant challenges in functioning. The significant negative loading for disconnection and rejection suggests that feelings of disconnection may contribute to positive social behaviors, preventing criminality. That for impairment of general functioning indicates that lack of functionality correlates positively with potential criminal behaviors, even among those with no prior records. This implies that fostering healthy relationships may mitigate crime risk.

In the group committing crimes against property, PC1 explains 52.24% of the variance and PC2 21.18% (Figure 3E). PC1 showed strong negative loadings for disconnection and rejection (−0.900), weakened autonomy (−0.850), defective borders (−0.773), targeting others (−0.833), and excessive vigilance (−0.875) (Supplementary Materials Table S6). Again, disconnection and rejection and excessive vigilance and inhibition appeared showed strong negative relationships, similar to the moderate-ADHD and severe-ADHD groups and non-criminals, suggesting a negative association with externalizing behaviors. Additionally, strong positive loadings in PC2 for disturbed interpersonal relationships (0.803) and impairment of general functioning (0.776) were again related to internalizing problems (Supplementary Materials Table S6). Disturbed interpersonal relationships had a positive loading, showing potential links to property crimes. Disconnection and rejection and excessive vigilance and inhibition both showed significant negative associations with property crimes, indicating that these factors may deter such behaviors among individuals with ADHD. The positive loading for disturbed interpersonal relationships suggests that relationship disturbances may increase the risk of property crimes. This suggests that problems in interpersonal dynamics can lead to property crimes, particularly if other supportive factors are compromised.

In the group of people convicted of crimes involving aggression, PC1 explained 53.38% of the variance and PC2 20.40% (Figure 3F). PC1 showed strong positive loadings for disconnection and rejection (0.909), defective borders (0.849), targeting others (0.797), and excessive vigilance (0.921) (Supplementary Materials Table S6). In contrast, disconnection and rejection and excessive vigilance and inhibition switched to strong positive loadings in this group, indicating their association with aggressive behaviors. This suggests that PC1 represents externalizing behaviors. PC2 showed strong positive loadings for disturbed interpersonal relationships (0.671) and impairment of general functioning (0.844), pointing to internalizing difficulties (Supplementary Materials Table S6). The variable of anxiety and depression showed moderate positive associations, reflecting emotional factors that may drive aggressive acts. Disconnection and rejection was significantly positive, indicating that feelings of disconnection may heighten aggressive tendencies. Excessive vigilance and inhibition strongly correlated with aggressive behavior, suggesting that anxiety and a heightened state of alertness lead to increased aggression. The finding for disturbed interpersonal relationships further supports the idea that relationship issues are strongly associated with aggressive tendencies in individuals with severe ADHD. Thus, feelings of rejection and anxiety can correlate with aggression, showing that emotional distress may provoke more severe criminal behaviors.

Across groups, PC1 consistently showed strong loadings (positive or negative) related to externalizing behaviors (defective borders, targeting others, weakened autonomy, disconnection and rejection, and excessive vigilance and inhibition). The operators (positive/negative) may change, but the involvement of these variables is apparent. On the other hand, across all groups, PC2 consistently showed strong loadings related to internalizing problems (anxiety and depression, disturbed interpersonal relationships, impairment of general functioning). The operators may vary, but these variables remain consistently associated.

The main difference lies in the operators of the loadings on PC1. The change in the operators of the loadings of externalizing variables on PC1 across different tables may reflect different underlying processes driving these behaviors and the relationships between externalizing and internalizing problems. The PCA suggested a complex relationship among ADHD severity, behavioral characteristics, and criminal behavior. The shifts in the prominence of different variables with changing ADHD severity and crime types indicate that various factors might be at play in each scenario. Further analysis and discussion are needed to understand these relationships’ nuances fully.

The patterns suggest that as ADHD severity increases, there is a shift in the prominent characteristics. In the negative-ADHD group (A), anxiety and depression are most strongly associated. As severity increases (B and C), attributes like defective borders, excessive vigilance and inhibition, and targeting others become more prominent. Disturbed interpersonal relationships also appears across severity levels, but its relative importance seems to change. Panels D, E, and F show the PCA for individuals with different criminal records. Similarly to the ADHD panels, the x and y axes represent PC1 and PC2, respectively, with percentages indicating variance explained.

This component often shows strong positive loadings for variables representing externalizing behaviors (defective borders, targeting others, weakened autonomy, disconnection and rejection, and excessive vigilance and inhibition). Conversely, it often shows weaker positive or negative loadings for internalizing problems (anxiety and depression, disturbed interpersonal relationships, impairment of general functioning). This suggests that PC1 broadly represents a dimension of behavioral disinhibition versus internalizing distress. The operator (+ or −) of the loadings for a specific variable could change from one table to another depending on the particular group, showing that PC1 is not necessarily a direct measure of externalizing behavior, but rather a combination of several factors. PC2 displays more variation across the tables. It often involves a contrast between variables representing internalizing and externalizing difficulties, but in a less systematic way compared to PC1. The loadings on PC2 are sometimes weaker than those on PC1, indicating that PC2 represents a less dominant dimension of variance.

Across all groups, the constructs of disconnection and rejection and excessive vigilance and inhibition consistently showed strong relationships with both severe ADHD and criminal behavior patterns. Disturbances in interpersonal relationships and impairments in general functioning were strong indicators across severity levels and crime types. As severity increases, the association with negative outcomes (anxiety, depression) strengthened, hinting at the need for integrated interventions targeting these issues across populations.

Disconnection and rejection and excessive vigilance and inhibition consistently appeared as critical factors. They correlated with emotional distress and interpersonal issues in ADHD. Impaired relationships are a significant theme in the crime-related tables. They often serve as a foundational element for protective factors against crime and risk factors for aggressive behaviors. The analysis showed a similar pattern of psychological and behavioral characteristics across the three groups, but with varying importance. Anxiety and depression and impairment of general functioning appeared consistently, but with shifting prominence concerning other characteristics like defective borders. The panel for crimes involving aggression (Figure 3F) shows the strongest association with defective borders and related characteristics.

The observations of correlations supported the analysis of the principal components. The correlation matrices illuminate the relationships between various psychological and behavioral characteristics and both ADHD severity and the presence/type of criminal behavior. Across all six groups (Supplementary Materials Tables S7 and S8), variables representing externalizing behaviors (disconnection and rejection, weakened autonomy and lack of achievement, defective borders, targeting others, excessive vigilance and inhibition) showed strong positive correlations with one another. This indicates that these behaviors tend to cluster together: individuals exhibiting one of these traits are more likely to exhibit others. The relationships between externalizing behaviors and internalizing symptoms (anxiety and depression, disturbed interpersonal relationships, impairment of general functioning) were generally weaker and less consistent across the groups. While some correlations existed, they were frequently not statistically significant (N/S), implying that the strength and direction of the association between externalizing behaviors and internalizing symptoms vary substantially across the different groups.

As ADHD severity increases from non-ADHD to severe, the intercorrelations among the externalizing behaviors generally remained high, but the magnitude showed some variation. The correlations between externalizing behaviors and internalizing symptoms changed. While internalizing problems may show some association with the externalizing behaviors in the lower-severity ADHD groups, the association appears less consistent and weaker at higher severity levels. This suggests a potential shift in the phenotypic presentation of ADHD across severity.

Comparing the three crime-related groups revealed a similar pattern to the ADHD severity groups. The no-crime group exhibited stronger correlations between externalizing and internalizing symptoms. In contrast, the crime-involving-aggression group demonstrated less pronounced and consistent correlations, suggesting that the co-occurrence of internalizing and externalizing traits is less intense in individuals engaging in aggressive criminal behaviors. The crime-against-property group again demonstrated an intermediate pattern. These relationships may indicate that individuals in the higher-severity ADHD groups and the crime-involving-aggression group exhibit less cohesion in their internal experiences and understanding of their own mental states. This in turn increases their tendency to rely on rigid, maladaptive coping mechanisms, such as outward-directed aggression or impulsive behaviors that lack a sufficiently defined direction or purpose.

The matrices suggest a core pattern of highly interconnected externalizing behaviors across all groups. The relationships between these externalizing behaviors and internalizing symptoms, however, were less consistent and appeared moderated by both ADHD severity and the type of criminal behavior. Individuals with more severe ADHD and those who engaged in aggressive criminal behavior seemed to display a somewhat stronger association between externalizing and internalizing problems. This implies that the clinical presentation of ADHD and its relationship to criminal behavior is multifaceted and likely involves complex interactions among different behavioral and psychological factors.

In the case of family and trauma, despite the results being similar in numbers, the vectors were primarily significant (in the case of ADHD and moderate ADHD, as well as in the absence of crime and property crime). However, regarding the lack of ADHD and severe ADHD, as well as the absence of crime and crimes involving aggression, there is general confusion about everything.

From a psychological perspective, if someone has experienced trauma and had a supportive family, they understand that it was inappropriate (as is the case with no ADHD and absence of crime). In the case of moderate ADHD, the family may have been supportive, but did not explain the traumatic event as negative: instead, they tried to divert attention. In contrast, in the case of severe ADHD and crimes involving aggression, the family sent ambiguous signals, meaning that for dramatic events, they negated both the child and the situation. Perhaps the family itself were aggressive, so the child had to rationalize that it was the norm—clearly, the signals were ambiguous.

## 4. Discussion

Mental and behavioral disorders have become a critical focus in the rehabilitation of incarcerated individuals. Research indicates that up to 70% of the incarcerated population may experience symptoms of mental disorders [2,3,4]. Among these, ADHD has emerged as a key area of investigation. While some studies suggest ADHD is a significant risk factor in criminal behavior [35,51,67], results remain inconclusive. For instance, a meta-analysis of long-term studies on ADHD and criminality found that hyperactivity was associated with a relative risk of 2.2 for arrests and 3.3 for convictions [39]. Other studies confirm a higher prevalence of hyperactivity among convicted individuals, including both men and women [32,39,68].

Conversely, some researchers caution against overinterpreting these findings. They argue that long-term studies often focus on clinical populations with pronounced ADHD symptoms or problematic behaviors [40]. Studies of incarcerated individuals further reveal that those with ADHD diagnoses frequently exhibit co-occurring mental disorders or socialization difficulties, both of which independently increase the risk of criminal behavior [51]. This suggests that ADHD alone may not significantly elevate the risk of criminality, but when coupled with other disorders, the likelihood increases substantially [24,52]. These discrepancies highlight the need for further investigation into factors that influence these relationships.

This study examined the relationships among family functioning, childhood trauma, early maladaptive schemas, mental health disorders, ADHD severity, and criminal behavior. The findings reveal complex interconnections among these factors. Traumatic events were prevalent across all groups analyzed, regardless of ADHD severity or criminal behavior. However, individuals without ADHD symptoms or a history of criminality reported greater family cohesion and a stronger protective influence of the family in coping with negative experiences. In contrast, this protective effect diminished as ADHD symptoms intensified, particularly among those convicted of violent crimes. Incarcerated individuals convicted of violent offenses described their family environments as less supportive and cohesive. These findings align with prior research highlighting the critical role of family dynamics in shaping children’s development and behavior [65,66].

The family environment, encompassing parents and primary caregivers, plays a pivotal role in daily interactions, communication, providing a protective space, and modeling social relationships. This aligns with meta-analyses showing statistically significant relationships between negative parenting practices and an increased likelihood of ADHD symptoms and diagnosis [67]. Similar patterns are observed in offender populations, where factors such as neglect, parental violence, or lack of support, when combined with biological vulnerabilities, contribute to repeated conflicts with the law [68,69].

The study also found a complex relationship among maladaptive emotional schemas, mental disorders, ADHD symptoms, and criminal behavior. In individuals without ADHD traits or criminal behaviors, a more precise distinction emerged between clinical symptoms of mental disorders (primarily anxiety and depression) and personality symptoms (manifested as maladaptive emotional schemas). However, as ADHD symptoms intensified and behaviors progressed from no criminal activity to property crimes and eventually to violent crimes, maladaptive emotional schemas became more pronounced. These schemas also showed stronger correlations with mental health symptoms.

Among individuals with more severe ADHD symptoms and violent criminal behavior, schema domains such as disconnection and rejection and excessive vigilance and inhibition were notably prevalent. Research confirms that maladaptive emotional schemas are associated with symptoms of psychological disorders, including anxiety and depression [70,71]. Consistently with these findings, the present study observed stronger associations among maladaptive emotional schemas, anxiety, and depression in groups with more intense ADHD symptoms and among those convicted of violent crimes. This suggests more significant psychological deficits in these groups regarding current symptoms and personality traits. Such deficits hinder the ability to form healthy interpersonal relationships or seek support from others.

ADHD and criminal behavior appear to be multifaceted phenomena influenced by individual (constitutional) and external (environmental) factors. Recognizing the disorder and providing a diagnosis is a crucial element of support, as pharmacological treatment alone has been shown to produce beneficial effects in managing observable symptoms of the disorder [72,73]. Environmental support is critical in mitigating the development of psychopathology, as it can buffer against the emergence of negative symptoms in adulthood. Individuals lacking adequate family support are more likely to struggle with negative experiences and are less capable of integrating life events positively. These individuals also exhibit a greater tendency to experience co-occurring mental disorders.

However, it is important to note that both this and other studies on environmental resources and their impact on rehabilitation and support for individuals with ADHD should be interpreted with caution, due to the specific nature of the studied populations.

These findings underscore the importance of carefully designed interventions for individuals with ADHD symptoms and offender populations. Such interventions should include individualized assessments to identify both resources and deficits. Programs should focus on teaching new, adaptive relationship patterns and, in line with schema therapy principles, fostering healthy role models who exemplify self-regulation, joy, and freedom.

Recognizing the critical role of the environment in providing support, modeling, and shaping appropriate attitudes, attention should also be given to the need for training and skill development among social services, public service officers, and healthcare professionals. Such efforts can help reduce the stigma associated with ADHD while simultaneously enhancing societal resources to promote socially beneficial attitudes.

These measures can empower individuals in these groups to better utilize societal resources and integrate more positively into their environments.

## 5. Limitations

This research has several limitations that should be taken into account. The tools used to assess ADHD relied on retrospective self-reporting requiring participants to recall past symptoms, which the researchers then categorized as indicative of the disorder. Due to the conditions of penitentiary isolation under which the study was conducted, it was impossible to verify these reported behaviors or assess their diagnostic significance.

The tool’s structure (DIVA-5.0), primarily based on interviews with the individual being assessed, further limits the ability to gather additional data. This methodological approach may contribute to phenomena such as impression management or self-deception. Consequently, the findings rely heavily on participants’ recollections and subjective perceptions of their past experiences.

In designing future studies, it would be valuable to consider collecting supplementary data from prison staff (medical and custodial) and exploring the possibility of gathering information from participants’ family environments.

Additionally, the study focused exclusively on male convicts serving sentences in lower-security facilities. While this group represents a significant portion of the incarcerated population in Polish prisons and is often the target of resource-based interventions (e.g., work programs), the findings may not be generalizable to other groups. For instance, the correlations observed in this study might be more pronounced in populations exhibiting higher levels of moral corruption.

Moreover, the exclusion of female participants limits the applicability of the findings to incarcerated women, who may experience different influences due to gender-specific factors such as the nature of crimes committed, upbringing, and societal expectations.

The cultural context of the study—Polish prisons—could be broadened to include other penitentiary environments, such as individuals serving substitute penalties or correctional facilities in other countries.

Finally, it also seems essential to design similar studies with a longitudinal approach. Examining both traumatic factors and family dynamics (potentially including intergenerational trauma) would provide a better understanding of the dynamics and interdependence of the relationships described in the manuscript. Such research could also significantly impact our understanding of the mutual influence of biological and environmental factors on the development of maladaptive emotional schemas associated with personality formation.

Future research should aim to include female participants and account for variability in correctional settings across different cultural contexts to enhance the generalizability of findings. Broadening the scope of the study to encompass these groups would offer a more comprehensive understanding of the relationships among ADHD, criminal behavior, and psychological factors.

## 6. Conclusions

The research presented is innovative, as it explores the intricate relationships among ADHD, criminality, and other psychological factors within a standardized sample of convicts exhibiting a lower degree of moral degradation. This cohort represents a substantial portion of the Polish prison population. The findings highlight the complex interplay among ADHD severity, criminal behavior, family environment, and psychological characteristics. They underscore the critical role of family support and early intervention in mitigating adverse experiences to reduce the risk of severe ADHD symptoms and associated criminal tendencies.

Clinical interventions should prioritize fostering supportive family environments, addressing maladaptive emotional patterns, and providing comprehensive care for internalizing and externalizing symptoms. Such an approach could help minimize the impact of ADHD on criminal behavior and improve outcomes for affected individuals. Within the context of this research, teaching family members specific skills related to providing support for individuals with ADHD seems particularly important. This is because properly directed support, providing both community and flexibility and allowing for emotional expression, enables the internalization of appropriate coping mechanisms for the future.

When diagnosing adults with hyperactivity traits, it is crucial to adopt a comprehensive approach that considers persistent symptoms across various domains, including relationships and mental health. This understanding would enable the development of targeted interventions to enhance functioning and support individuals in adopting adaptive coping mechanisms for past traumas.

For adults with hyperactivity traits in penitentiary settings, additional support in managing deficits, mental health difficulties, and family and social relationships is crucial. Consequently, it is worthwhile to train prison staff to understand the nature of ADHD and its consequences, as well as the possibilities of providing appropriate support. Simultaneously, it is essential to limit the phenomenon of stigmatization, which could hinder individuals with ADHD from serving their sentences in conditions with a reduced security system. Tailored interventions can empower them to navigate their challenges more effectively and reduce the likelihood of recidivism.

## Figures and Tables

**Table 1:** Sociodemographic data of the participants.

	Category	*n*	%
Severity of ADHD	Non-ADHD	303	86
	Moderate ADHD	33	9
	Severe ADHD	16	5
Nature of crime	No criminal record	118	34
	Against property	193	55
	Involving aggression	41	12
Age (years)	Mean ± standard deviation	38 ± 10 years	
Marital status	Single	212	60
	Married	79	22
	Divorced	58	16
	Widowed	3	1
Education	Primary	55	16
	Vocational	84	24
	Secondary	146	41
	Bachelor’s degree	15	4
	Higher	52	15
Mother’s education	Primary	42	12
	Vocational	103	29
	Secondary	130	37
	Bachelor’s degree	4	1
	Higher	73	21
Father’s education	Primary	38	11
	Vocational	139	40
	Secondary	117	34
	Bachelor’s degree	3	1
	Higher	50	14
Family	Complete	239	68
	Incomplete	69	20
	Reconstructed	44	13
Family upbringing	Mother only	179	51
	Father only	13	4
	Equal parents	135	38
	Grandparents	22	6
	Other	3	2
Mental illness in family	No	321	91
	Yes	31	9
ADHD in childhood	No	341	97
	Yes	11	3

The mean age and standard deviation in groups for ADHD were: non-ADHD 38 ± 10.2 years, moderate ADHD 34 ± 7.4 years, and severe ADHD 38 ± 10 years. There were no significant differences in age between ADHD groups (*p* = 0.0845); Nature of crime: no criminal record 33 ± 10.1 years, against property 40 ± 9.6 years, and involving aggression 38 ± 7.8 years. There was a significant difference in age between the crime-dependent groups (*p* < 0.0001): the group of participants without criminal records was significantly younger than both other groups with criminal records, but there were no significant differences regarding age of the groups with criminal records.

## Data Availability

The raw data supporting the conclusions of this article will be made available by the authors on request.

## Supplementary Materials

The following supporting information can be downloaded at https://www.mdpi.com/article/10.3390/brainsci15020141/s1. Supplementary correlation matrices and eigenvectors for analyzed data. Table S1: ADHD eigenvectors; Table S2: CRIME eigenvectors; Table S3: ADHD correlations; Table S4: CRIME Correlations; Table S5: ADHD eigenvectors A; Table S6: CRIME eigenvectors A; Table S7: ADHD correlations A; Table S8, CRIME Correlations A.
